# Acromegaly in the elderly patient

**DOI:** 10.20945/2359-3997000000194

**Published:** 2019-11-01

**Authors:** Raquel S. Jallad, Marcello D. Bronstein

**Affiliations:** 1 Hospital das Clínicas Faculdade de Medicina Universidade de São Paulo São Paulo SP Brasil Unidade de Neuroendocrinologia, Serviço de Endocrinologia e Metabologia, Hospital das Clínicas, Faculdade de Medicina, Universidade de São Paulo, São Paulo, SP, Brasil

**Keywords:** Acromegaly, elderly, somatostatin, receptor ligands, pituitary, mortality

## Abstract

Acromegaly is an insidious disease, usually resulting from growth hormone hypersecretion by a pituitary adenoma. It is most often diagnosed during the 3rd to 4th decade of life. However, recent studies have shown an increase in the incidence and prevalence of acromegaly in the elderly, probably due to increasing life expectancy. As in the younger population with acromegaly, there is a delay in diagnosis, aggravated by the similarities of the aging process with some of the characteristics of the disease. As can be expected elderly patients with acromegaly have a higher prevalence of comorbidities than younger ones. The diagnostic criteria are the same as for younger patients. Surgical treatment of the pituitary adenoma is the primary therapy of choice unless contraindicated. Somatostatin receptor ligands are generally effective as both primary and postoperative treatment. The prognosis correlates inversely with the patient’s age, disease duration and last GH level. Arch Endocrinol Metab. 2019;63(6):638-45

## INTRODUCTION

Increased life expectancy has become a reality in recent decades, leading to an aging population ( 1 ). Like other diseases, prevalence of acromegaly in older patient (>65 years) has also increased ( 2 - 7 ). Untreated acromegaly is associated with reduced life expectancy ( 8 - 10 ). Accuracy and effectiveness in diagnosis, treatment and control of risk factors are important in order to minimize impact of acromegaly in patient’s expected lifespan, as well as to contribute to better quality of life and reduced morbid-mortality.

## GROWTH HORMONE AND AGING

Pituitary growth hormone (GH) secretion is pulsatile and controlled mainly by two hypothalamic peptides: GH-releasing hormone and somatostatin, which respectively stimulates and inhibits its release. GH induces the generation of insulin-like growth factor 1 (IGF- 1 ) in the liver and regulates the paracrine production of this peptide in many other tissues. By its turn IGF-1 feedbacks to the hypothalamus and modulates somatostatin release, thus inhibiting further GH synthesis and secretion ( 11 ).

GH secretion varies throughout life, reaching a peak in adolescence and then declining with age in both men and women ( 11 ). Between 18 and 30 years of age, there is an exponential decline in the levels of GH and its downstream effector IGF-1, reaching very low levels in individuals over sixty years of age ( 11 ). The levels of GH and IGF-1 in old age overlap those of younger adults with classical GH-deficiency, and many age-associated changes resemble those of GH-deficiency ( 12 , 13 ). The decline in GH secretion during normal aging by approximately 14% per decade ( 12 , 14 ) can be attributed to several potentials age-related mechanisms such as decreased GHRH and/or ghrelin secretion ( 15 ), increased somatostatinergic tone, reduced pituitary response to GHRH or ghrelin and increased sensitivity to negative IGF-1 feedback ( 13 - 15 ). These age-related somatic and psychological changes became known as “somatopause” ( 12 , 16 , 17 ). whose physiological meaning has not yet been clarified. This has prompted speculation that decline in the somatotropic axis may be a “protective” effect concerning highly prevalent morbidities in aging individuals, as diabetes mellitus, arthritis and neoplasms.

In acromegaly, the GH rhythmicity, sexual dimorphism of GH rhythm and age-related decline of GH secretion are preserved in patients with high GH values ( 18 ). These finding point that the GH hypersecretion by the pituitary adenoma is not entirely autonomous, but is still subject to the normal hypothalamic regulation. However, most patients with acromegaly have elevated random GH levels that do not suppress upon glucose administration ( 18 ). In the elderly, this central regulation of GH secretion is similar to younger patients.

## EPIDEMIOLOGY

Acromegaly is a relatively rare disease caused by long-standing GH hypersecretion. According to recent epidemiologic data, the estimated prevalence and annual incidence of acromegaly are 18-137 and 2-11 cases per million person-year, respectively ( 2 - 7 ). The diagnosis of acromegaly is usually made during the 3rd to 4th decade of life ( 2 - 7 ). Recent studies have shown an increase in the incidence and prevalence of acromegaly in the elderly, probably due to increasing life expectancy ( 2 - 7 ). However, in elderly patients, disease remains a rare condition, even with increased diagnosis rates. According to recent data from the European Study involving 14 centers encompassing almost 3,200 patients with acromegaly, diagnosis of individuals over 65 years became more frequent, probably as a result of increase in life expectancy associated with better awareness of the disease, even in patients with mild features. Therefore, the delay in diagnosis and treatment has been reduced ( 7 ).

A recent American study showed that both incidence and prevalence of acromegaly had increase in older patients, with no difference between genders ( 3 ). Interesting enough, the annual incidence and prevalence in adults 65 years and older were estimated in 9 to 18 cases per million person-years and 148 to 182 cases per million in adults, respectively ( 3 ).

In women, the delay in diagnosis may be due to the protective role of estrogen, reducing IGF-1 levels before menopause ( 19 , 20 ).

## CLINICAL MANIFESTATIONS

The onset of signs and symptoms is slow in the majority of cases, with typical facial changes, arthralgias, asthenia, paraesthesia and sensation of enlargement of the lower limbs more subtle than in younger patients ( 21 , 22 ). The clinical picture may be confused with the features observed during normal aging ( 23 - 25 ).

Among the surgical series, the prevalence of symptoms of tumor mass effect varies from 50 to 80% of cases and usually leads to the diagnosis of acromegaly. Sometimes visual abnormalities may be confused with symptoms of age-related eye diseases such as cataracts, macular degeneration, and vascular eye diseases ( 25 , 26 ).

Elderly patients with acromegaly have a higher prevalence of comorbidities than younger patients ( 23 - 25 , 27 ). The exact prevalence of the comorbidities is unknown. The estimated prevalence rates show a wide variability, that can reflect the different populations studied and the different comorbidities diagnostic criteria in the studies. It is also important to note that the indices are not derived from epidemiological studies. Age itself is a confounding factor for estimated rates, since in the elderly population without acromegaly the prevalence of hypertension and diabetes mellitus is higher than those observed in younger normal individuals ( 23 - 25 ). The high prevalence of comorbidities in older individuals also contributes to the delay in acromegaly diagnosis. In fact, changes in carbohydrate metabolism and hypertension in the elderly can be considered as related to aging by itself, and therefore the acromegaly diagnosis may be missed ( 25 , 28 ).

Studies evaluating the presence of comorbidities only in elderly patients with acromegaly are scarce ( 21 - 27 , 29 ) data are shown in Table 1 .

**Table 1:** Clinical and endocrine parameters in the elderly patients

	N	Mean age/range (years)	Sex (female/male)	GH (ng/mL)	IGF-1 (ng/mL)	Macro (%)	DM (%)	HT (%)	Cardiac/vascular disease (%)	Neoplasy (%)
Puchner et al., 1995^21^	15	68.3/65-81	13F/2M	47.4	1,112	73	27	60	7	NA
Minniti et al., 2001^22^	22	68.5 /66-74	11F/11M	21.5	556	82	41	50	NA	NA
Colao et al., 2007^23^	57	70.0 /68-71	27F/30M	30.8	598	70	58	82	29.2	NA
Tanimoto et al., 2008^25^	16	NA/61-82	10F/6M	6.7	740	63	63	53	NA	67
Arita et al., 2008^26^	9	73.2/70-82	3F/4M	29.4	610	89	86	71	29	55
Dupuy et al., 2009^27^	68	76.8/70-95	46F/22M	6.7	439	85	42	80	10	38-46
Sasagawa et al., 2018^29^	24	68.0/65-75	18F/6M	7.1	456	33	46	63	7	8

GH: growth hormone; IGF-1: insulin-like growth factor-1; Macro: macroadenoma; DM: diabetes mellitus; HT: hypertension; NA: not available.

**Figure 1:**
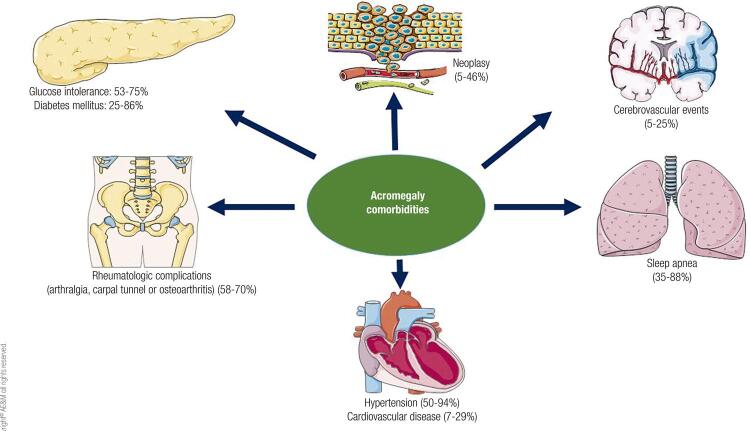
Comorbidities and its prevalence data in elderly patients with acromegaly.

Figure 1 also includes the prevalence of comorbidities in elderly patients extracted from general acromegaly multicentric registers ( 5 , 9 , 27 , 30 - 33 ).

Hypertension plays a significant role in the development of cardiac hypertrophy, especially in older acromegalic patients and diastolic blood pressure is best predictive factor for cardiac hypertrophy ( 24 , 27 ). Therefore, early and aggressive treatment of hypertension is essential in order to minimize cardiovascular disease in acromegaly.

In older patients, congestive heart failure as end-stage acromegalic cardiomyopathy occurs more frequently ( 23 ). It is suggested that aging and long duration of exposure to elevated GH/IGF-I levels are key determinants of cardiac abnormalities ( 31 , 34 ).

GH hypersecretion leads to insulin resistance ( 35 ). In acromegaly, the estimated prevalence of diabetes is higher in patients over 65 years than in younger patients (27%-86% *vs.* 19%-56%) ( 29 ). Based on literature data, the most characteristic predisposing factors for diabetes were older age, longer duration of illness and family history of diabetes ( 23 , 32 ). Diabetes is also associated with abnormalities in lipid metabolism, such as a tendency towards high cholesterol levels and hypertriglyceridemia. Not all studies have identified these outcomes ( 32 ). Acromegaly control often leads to improvement of insulin resistance and improvement of diabetes ( 32 ).

Obstructive sleep apnea syndrome is a common disorder in patients with acromegaly, and also specifically associated with increased mortality ( 36 ). The prevalence of sleep apnea in acromegaly ranges from 45% to 80% ( 33 , 37 ). Interesting enough, in elderly patients the prevalence seems to be similar to younger ones ( 33 , 37 ).

The frequency of rheumatologic complications (arthralgia, carpal tunnel or osteoarthritis) does not seem statistically different from the elderly population without acromegaly ( 27 ). This finding may be explained by the prevalence of these complaints in the general elderly population, as well as by the inaccuracy of questionnaires addressing this issue.

A recent study evaluated the presence of cognitive and functional alterations in elderly patients with acromegaly and in elderly in the general population ( 38 ). Elderly with acromegaly exhibited a higher frequency of impaired cognitive functions, reduced mobility, difficulty in performing daily activities, and dementia, as compared with their counterparts without acromegaly. In addition, elevated GH/IGF-1 levels and longer duration of acromegaly tended to positively correlate with these changes ( 38 ).

GH and IGF-I hypersecretion are associated to development and progression of malignancies in patients with acromegaly, even so this remains a controversial issue ( 39 ). A recent meta-analysis revealed a moderate increased cancer risk, but mainly observed in single center studies ( 10 ). In controlled acromegaly, the cancer mortality is comparable to the one observed in the general population ( 10 , 40 ). On the basis of recent study, in the last decade, there were a significant decrease in mortality and improvement in life expectancy in patients with controlled acromegaly, especially in patients treated with SRL’s. It resulted in change in most common cause of death in acromegaly and cancer became the most common cause of mortality in these patients, just as in the general population ( 41 ). In the few studies evaluating this issue in elderly with acromegaly the estimate prevalence rates appear to be similar to those observed in younger patients ( 21 , 23 , 26 , 27 , 29 ) – Table 1 .

## DIAGNOSIS

The diagnostic and remission/disease control criteria are similar to those used for younger patients ( 42 - 44 ).

According the recent guidelines, the clinical suspicion of acromegaly is confirmed by the demonstration of autonomous GH hypersecretion and elevated serum IGF-1 levels according to age ( 42 - 44 ). Due to the pulsatility and the short half-life of GH, acromegaly diagnosis by single random GH levels may be misleading ( 45 ). In contrast, due to its relatively stable levels and long half-life IGF-1 can be used for acromegaly screening. Therefore, normal IGF-1 levels adjusted-age effectively excludes the diagnosis of acromegaly. However, some conditions may determine low IGF-1 levels as chronic diseases as uremia, hepatic failure and uncontrolled diabetes mellitus, poor nutritional status and advanced age ( 45 ). In these cases, lack of suppression of GH to less than 1 μg/L or 0.4 μg/L during an oral glucose tolerance test (OGTT) points to acromegaly diagnosis ( 42 , 43 ). Nevertheless, OGTT is not indicated for patients with diabetes mellitus ( 42 ).

As matter of fact, no special considerations about acromegaly diagnosis in aged patients are placed in guidelines ( 42 - 44 ). Moreover, the concept of lower GH/IGF-1 levels in the elderly is not a consensus in the literature ( 21 , 22 , 25 , 26 , 29 ).

Following biochemical diagnosis, contrast enhanced magnetic resonance imaging (MRI) of the sellar region is required to assess tumor size, localization and invasiveness ( 42 ). Gadolinium enhancement should however be used with caution or be avoided in patients with renal impairment. If MRI is contraindicated or unavailable, pituitary computerized tomography should be performed ( 42 ).

Despite some data pointing to smaller tumors in older acromegaly population ( 46 ), this finding is not confirmed by most studies ( 21 , 22 , 26 ) – Table 2 .

**Table 2:** Surgical and endocrinologic outcomes of patients with acromegaly over 65 years

	N	Mean age/range (years)	GH (ng/mL)	IGF-1 (ng/mL)	Macro (%)	ASA-PS ≥ 2 (%)	Remission rate (%)	Perioperative complication (%)	Mortality (%)
Puchner et al., 1995^21^	15	68.3/65-81	47.4	1,112	73	86	27	None	0
Minniti et al., 2001^22^	22	68.5/66-74	21.5	556	82	73	68	13.7	0
Arita et al., 2008^26^	9	73.2/70-82	29.4	610	89	100	72	None	0
Sasagawa et al., 2018^29^	24	68.0/65-75	7.1%	456	33	100	67	17%	0

GH: growth hormone; IGF-1: insulin-like growth factor 1; Macro: macroadenoma; ASA-PS: American Society of Anesthesiologists Physical Status; macroadenoma; Remission Rate GH < 1.0 ng/mL and normal IGF-1.

## PATHOLOGY

Acromegaly is almost always (98%) caused by a pituitary tumor, either a somatotroph adenoma or a mixed somatotroph-lactotroph adenoma. Based on histopathological evaluation, the somatotropinoma is classified as densely granulated somatotroph adenoma (DGSA) and sparsely granulated somatotroph adenoma (SGSA) ( 47 ). The DGSA is the most frequent subtype and its pathological features confer better overall prognosis ( 47 , 48 ). They are smaller, easier to remove, and less frequently recur after surgery ( 47 , 48 ). Usually, they have higher somatostatin receptor subtype 2 (SST 2 ) protein expression than SGSA with consequent better response to first-generation long-acting somatostatin receptor ligand (SRL) therapy ( 49 , 50 ). Moreover, DGSA usually present hypointensity in T2-weighted MRI, which may be used as a predictor of response to SRL’s ( 51 - 54 ). Comparing to SGSA, patients harboring DGSA have higher age at diagnosis and exhibited longer disease duration before diagnosis, suggesting milder symptomatology ( 48 ). This study ( 48 ) supports the hypothesis that in elderly patients, the DGSA subtype is the most frequent. Nevertheless, more data on this issue is needed.

## TREATMENT

The overall goals of treatment in older patients are similar to those of younger ones and include management of both acromegaly (hormonal control and tumor reduction) and common geriatric medical conditions.

In acromegaly, the effectiveness of surgical treatment correlates inversely with preoperative GH and IGF-I levels and tumor size ( 55 ).

The available treatment modalities are transsphenoidal surgery, medical therapy and radiotherapy.

### Pituitary surgery

Usually, pituitary surgery by the transsphenoidal approach is the first treatment modality also in the elderly, with relatively low risk of morbidity ( 13 , 24 ) and high probability of remission, especially in microadenomas and enclosed macroadenomas ( 13 , 24 , 26 ). Due to the physiological process of aging, the elderly are particularly susceptible to stress of hospitalization, anesthetic agents and surgery. To aggravate the vulnerability of this population, acromegaly is often associated with hypertension, cardiovascular disease and diabetes. This vulnerability translates into an increased morbidity and mortality seen in elderly as compared to younger patients ( 29 ). Surgical series applied the surgical risk classification of the American Society of Anesthesiologists to investigate the role of the preoperative physical state as a possible predictor of complications in elderly with acromegaly ( 56 ). In these series most of patients showed ASA grade ≥ 2 ( 21 , 22 , 26 , 29 ). Comparing to younger patients, elderly ones did not show a higher incidence of perioperative complications. This results might be related to rigorous perioperative care: 1 – preoperative medical treatment optimization, to avoid postoperative complications; 2 – adequate anesthesia care; 3 – careful surgical technique and 4 – proper postoperative management, even in patients who achieved endocrinologic remission. In line with this findings, about 50% of cases after 70 years and almost 90% under 70 years underwent surgery as primary treatment ( 21 , 22 , 26 , 29 ). Therefore, endonasal transsphenoidal surgery with good anesthetic-, peri-, and postoperative management is a safe and efficient treatment for selected elderly with acromegaly, patients with adequate clinical conditions and/or presence of visual adverse events. Additionally, the probability of substantial tumor removal should also be taken into account ( Table 2 ).

### Medical treatment

Current pharmacological therapy options are somatostatin receptor ligands (SRL’s), dopamine agonists and the GH receptor antagonist, pegvisomant.

Although medical therapy is more often reserved for patients awaiting surgery or with persistent disease postoperatively, primary medical therapy should be indicated for: patients with high risk of perioperative complications, those with limited life expectancy (<2 years) due to the severity of some comorbidities and when the tumor is not expected to be removed.

In patients with mild disease and slightly elevated IGF-1 levels, dopamine agonist, cabergoline, may be considered as initial therapy. In a study with small numbers of patients using dopamine agonist, most patients showed declines of GH-plasma levels, but not reaching normalization. In addition, the presence of adverse events led to discontinuation of treatment in several patients ( 21 ).

Generally, treatment with first-generation SRL’s (octreotide-LAR and lanreotide Autogel) leads to hormonal control of acromegaly in about 40% of patients ( 57 ). Elderly acromegaly patients, particularly male, showed higher hormonal responsiveness to octreotide than younger patients ( 58 ). This data reinforces the indication of SRL’s as the medical treatment of choice in elderly patients. However, this therapy may be associated with side effects, which should be monitored. The treatment may be continued for a long time for responsive patients without severe adverse events. The somatostatin receptor multiligand pasireotide-LAR is an option for patients resistant to first-generation SRL’s. Studies that included older patients with acromegaly showed its efficacy on hormonal control ( 59 , 60 ). Nevertheless, hyperglycemia is an adverse event frequently reported during pasireotide therapy, hence close monitoring of blood glucose levels and proper diabetes control is mandatory during treatment with this medication.

Concerning pegvisomant, clinical studies did not encompass significant number of subjects age over 65 years, but did not present geriatric-specific problems, that could limit the usefulness of pegvisomant in the elderly population ( 61 , 62 ). Nevertheless, elderly patients are more likely to age-related heart, liver, or kidney problems, requiring dose adjustment of pegvisomant.

There is some conflicting evidence that pre-surgical medical therapy may improve surgical outcome ( 63 ).

### Radiotherapy

In general, radiotherapy has been indicated as third line treatment for acromegaly, particularly in patients not controlled by previous surgery and/or medical treatment ( 42 , 44 ). In the elderly, this option seems attractive mainly due to lack of serious side effects. However, the delay on hormonal normalization, that occurs on average 5 to 10 years after irradiation, may limit its indication. Moreover, it is worth noting that radiotherapy is associated with high risk of pituitary insufficiency, requiring hormone replacement therapy, and a potential risk of cerebrovascular diseases ( 64 ).

A recent retrospective multicenter study evaluated prognostic factors of long-term efficacy, and tolerability of gamma knife radiosurgery for acromegaly ( 65 ). On univariate and multivariate analysis for prognostic factors affecting endocrine remission, no significant difference was observed regarding age: older patients did not show better endocrine control than younger ones.[65] This findings clash with the notion that elderly patients are more sensible to radiation effects than younger patients ( 66 ).

Based on data reported in large retrospective studies and systematic reviews young and older patients show similar local control and toxicity after either radiosurgery or fractionated stereotactic radiotherapy ( 65 , 67 - 69 ). Single-fractions doses of 20-28 Gy are usually employed for acromegaly ( 65 , 67 - 69 ).

## CONCLUSION

Acromegaly is rarely diagnosed in the elderly and can be a cause of mortality due to systemic complications, mainly cardiovascular diseases. Clinical picture may be confounded with features associated with aging and therefore the diagnosis may not be performed or delayed. In these patients, a multidisciplinary approach, preferable in a Pituitary Center of Excellence may be required to control the disease and reduce its morbidity ( 70 ). Although surgery remains the first treatment approach for most cases, SRL’s are a key medical therapeutic tool. In cases that do not adequately respond, the GH receptor antagonist pegvisomant or radiotherapy are additional tools for controlling IGF-I levels.
